# Vidjil: A Web Platform for Analysis of High-Throughput Repertoire Sequencing

**DOI:** 10.1371/journal.pone.0166126

**Published:** 2016-11-11

**Authors:** Marc Duez, Mathieu Giraud, Ryan Herbert, Tatiana Rocher, Mikaël Salson, Florian Thonier

**Affiliations:** 1 Université de Lille, CNRS, UMR 9189 – CRIStAL – Centre de Recherche en Informatique Signal et Automatique de Lille, 59000 Lille, France; 2 Inria Lille, 59650 Villeneuve d’Ascq, France; 3 School of Social and Community Medicine, University of Bristol, Bristol, United Kingdom; 4 SIRIC ONCOLille, 59000 Lille, France; 5 Inserm, Hôpital Necker – Enfants Malades, 75015 Paris, France; Western University, CANADA

## Abstract

**Background:**

The B and T lymphocytes are white blood cells playing a key role in the adaptive immunity. A part of their DNA, called the V(D)J recombinations, is specific to each lymphocyte, and enables recognition of specific antigenes. Today, with new sequencing techniques, one can get billions of DNA sequences from these regions. With dedicated Repertoire Sequencing (RepSeq) methods, it is now possible to picture population of lymphocytes, and to monitor more accurately the immune response as well as pathologies such as leukemia.

**Methods and Results:**

Vidjil is an open-source platform for the interactive analysis of high-throughput sequencing data from lymphocyte recombinations. It contains an algorithm gathering reads into clonotypes according to their V(D)J junctions, a web application made of a sample, experiment and patient database and a visualization for the analysis of clonotypes along the time. Vidjil is implemented in C++, Python and Javascript and licensed under the GPLv3 open-source license. Source code, binaries and a public web server are available at http://www.vidjil.org and at http://bioinfo.lille.inria.fr/vidjil. Using the Vidjil web application consists of four steps: 1. uploading a raw sequence file (typically a FASTQ); 2. running RepSeq analysis software; 3. visualizing the results; 4. annotating the results and saving them for future use. For the end-user, the Vidjil web application needs no specific installation and just requires a connection and a modern web browser. Vidjil is used by labs in hematology or immunology for research and clinical applications.

## Introduction

The immunological diversity of the lymphocytes mainly comes from the V(D)J recombinations. These recombinations are also useful markers of pathologies, and in leukemia, are used to quantify the minimal residual disease (MRD) during patient follow-up [1]. High-throughput sequencing (HTS) now enables the deep sequencing of a lymphoid population, analyzing and quantifying the *clones* that could come either from an immune response or from a pathology. Dedicated Repertoire Sequencing (RepSeq) methods and software [2] are necessary to deal with the specificity of V(D)J recombinations, that is handling small recombinations, somatic hypermutations, and short insertions.

Many tools for the in-depth analysis of V(D)J recombinations were developed by IMGT [3, 4]. Recently, new software able to deal with up to millions of sequences have appeared: [5], IgBlast [6], Decombinator [7], miTCR [8], TCRKlass [9], MiXCR [10], IMSEQ [11]. At the heart of these programs is optimized comparison of the reads against germline databases to detect and quantify *clonotypes*, whose definitions are supposed to overlap as much as possible the definition of biological clones. IgGalaxy has the purpose to provide an easy-to-use interface to IgBlast and IMGT/HighV-QUEST results [12]. Some other programs enable to further analyze or visualize statistics on the whole lymphocyte population, such as ARResT/Interrogate [13], tcR [14], ImmunExplorer [15] or VDJviz [16].

The programs that are only focused on the algorithmics part are difficult to use for biologists or clinicians. There is the need to have *easy-to-use interfaces for labs without bioinformatics expertise*, suitable for daily clinical or research job in RepSeq studies, allowing to easily navigate between statistical informations on the clonotypes and detailed view of some sequences and enabling user annotations. Some software try to fill this gap, such as IgGalaxy. However it relies on software that are not specifically suited to the analysis of high-throughput sequencing (with millions of sequencing reads). Also the user needs to upload the results from IMGT/HighV-QUEST, together with the sequence files, which makes the use of the software less practical. ARResT/Interrogate allows to upload results in tabular form (obtained, for instance, with IMGT/HighV-QUEST), and to display results and statistics. However, it does not provide easy sequence upload and processing, handling of large databases of patients or experiments and storage of user annotations.

**Fig 1:**
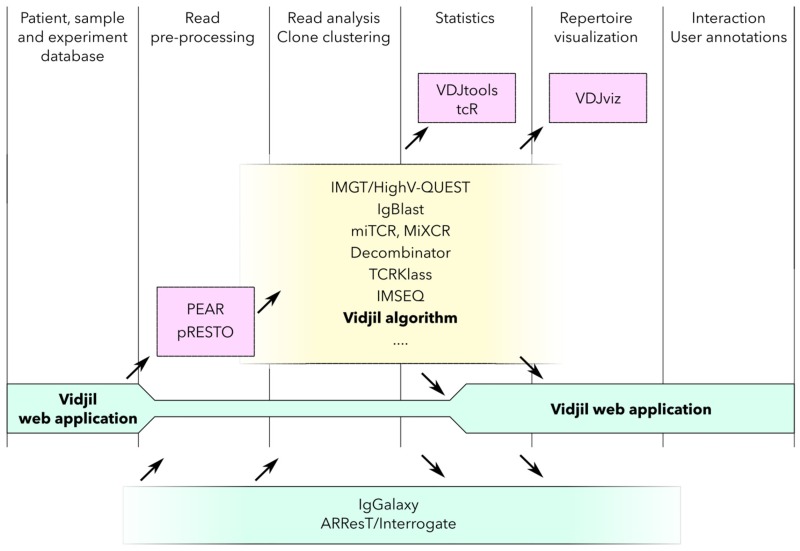
Repertoire Sequencing (RepSeq) analysis software generally take as input a set of reads and process this set analyzing V(D)J recombinations and gathering them into clonotypes while computing statistics on the repertoire. Some of these software further include pre-processing as well as visualization capabilities. Finally, some more specialized software focus on specific aspects of RepSeq studies. The originality of the Vidjil platform is to propose a complete pipeline for the end-user, starting from the raw reads to the interactive analysis. The Vidjil web application currently runs the Vidjil algorithm, MiXCR, PEAR, and has links to IMGT/V-QUEST and IgBlast. Further software integration is planned. Note that IgGalaxy is also built on a pipeline concept that allows to pipe several software. A key feature of the Vidjil web application is the sample, patient and experiment database, accessible from the web application client, that allows a daily clinical or research use without bioinformatics knowledge. Moreover, the client of the Vidjil web application can also be used independently to interact with the results of a RepSeq analysis.

We present here the Vidjil platform, that is ready for hospital or research lab use. The platform is made of efficient algorithms, a flexible web application consisting in the visualization and annotation of one or several samples, as well as a database storing valuable informations on samples, patients and experiments. The whole platform enables an autonomous usage in an immunology or hematology lab. This platform has a much wider spectrum than the original Vidjil algorithm described previously [17], which only consisted of a C++ program on the command-line to analyse high-throughput sequencing reads on TR*γ* and IgH loci. The algorithm now processes all immunoglobulin and T-cell receptor human loci, as well as some incomplete or unusual rearrangements. Moreover, we now offer a web application, which displays the results, stores the data and runs the analyses using several software: The platform proposes indeed several ways to analyze data with complementary software (at the moment: IMGT/V-QUEST, IgBlast, Blast, MiXCR). To our knowledge, Vidjil is the first open-source RepSeq platfrom enabling this autonomous usage, from raw sequence files to analysis, annotation and storage (Fig 1).

## Design and Implementation

The Vidjil platform can run any RepSeq program thats outputs V(D)J clonotypes from input data. Even if the platform was initially designed for the Vidjil algorithm, it does not rely on a specific algorithm: It includes other software, as for example MiXCR (see in the clinical data analysis, below). The following sections describe both the updated algorithm and the client and server sides of the web application. Vidjil is developed with systematic testing (more than 2,000 tests targeting all components, algorithm, web application client and server), continuous integration and regular releases (see S2 File).

### High-throughput Algorithm

The Vidjil algorithm, implemented in C++, processes high-throughput sequencing data (.fasta, .fastq, or .gz compressed files). Through a seed-based method, it detects sequences with V(D)J recombinations and gather them into clonotypes [17]. The key idea is that the clustering is done on a 50 bp nucleotide sequences at the V(D)J junction, and the detailed V(D)J assignation is done *after* the clustering. This makes the analysis extremely fast because, in the first phase, no alignment is performed.

**Fast clustering of recombined sequences.** Words of length *k* (the *k*-mers, with *k* ranging from 9 to 13, possibly with additional *don’t-care* characters) corresponding from V and J regions are detected on each read, allowing to locate a “window” overlapping the actual CDR3. The reads are gathered according to this window, and the algorithm also computes clonality measures to assess the diversity of samples: Shannon’s diversity *H*, Shannon’s equitability *E* and Simpson’s diversity *Ds*.

The algorithm was initially applied on TR*γ* and IgH loci [17]. It was extended to have an as complete as possible analysis of lymphoblast and lymphocyte sequences arising from all stages of the human hematopoiesis. Indeed, the algorithm now analyzes reads recombined from all immunoglobulin (IgH, Ig*λ*, Ig*κ*) and T-cell receptor human loci (TR*α*, TR*β*, TR*γ*, TR*δ*), as well as some incomplete or unusual rearrangements (Dh/Jh, D*δ*2/D*δ*3, KDE-Intron, mixed TR*α*-TR*δ* recombinations), by looking for *k*-mers corresponding from given “left” (5’) and “right” (3’) regions.

Some incomplete recombinations start from a D gene. As D genes are very short (8 to 37 bp), there may be not enough *k*-mers to detect them, especially when the recombination added mutations or deletions. An improvement was to include neighbor regions of the germline genes: for example, D*δ*2-J*δ* recombinations usually contain some sequence upstream of the D*δ*2 gene.

The germline genes are taken from IMGT/GENE-DB [18], that are free for academic use. Other genomic regions (KDE, Intron, neigbhbor genes) are obtained by direct queries to GenBank (ncbi.nlm.nih.gov). The program can be configured to look for other recombinations and can work with any germline file or combination of germline files, through configuration in the germlines.data file described in the documentation, for instance to analyze sequences from other species.

**Detailed analysis of clustered clonotypes.** Once reads have been gathered into clonotypes, the detailed V(D)J designation is computed by dynamic programming. Now the algorithm also detects some VDDJ or VDDDJ recombinations that may happen in the TR*δ* locus. Finally, the algorithm includes a CDR3/JUNCTION detection based on the position of Cys104 and Phe118/Trp118 amino acids. This detection relies on alignment with gapped V and J sequences, as for instance, for V genes, IMGT/GENE-DB sequences [18].

**Tests on curated sequences.** Our test case includes carefully curated sequences containing V(D)J recombinations. The curator (collaborators in the Lille hospital, at the Necker hospital in Paris or at the GOSH hospital in London) analyzed some unusual sequences by hand and possibly with different software. He then specified the precise designation of V, D and J genes that he would expect to see [19]. Those tests are designed so that no bioinformatics knowledge is needed. The tests are written in seemingly .fasta files containing the raw DNA sequences. The headers of the sequences specify the V(D)J designation of the sequence as well as the locus it belongs to. The specification of the format is available at vidjil.org/curated-vdj.

### Web Application for Interaction with RepSeq Data

The Vidjil web application, developed in Javascript with jQuery and d3.js, is made for the visualization, inspection and analysis of clonotypes and their tracking along the time in a MRD setup or in a immunological study. The web application visualizes data processed by the Vidjil algorithm or any RepSeq clonotype gathering software as soon as they output a compliant JSON format (documented on vidjil.org/doc). This gives some modularity to users if they need to combine Vidjil results with other data, coming from either personal analysis or other software or scripts.

**Fig 2:**
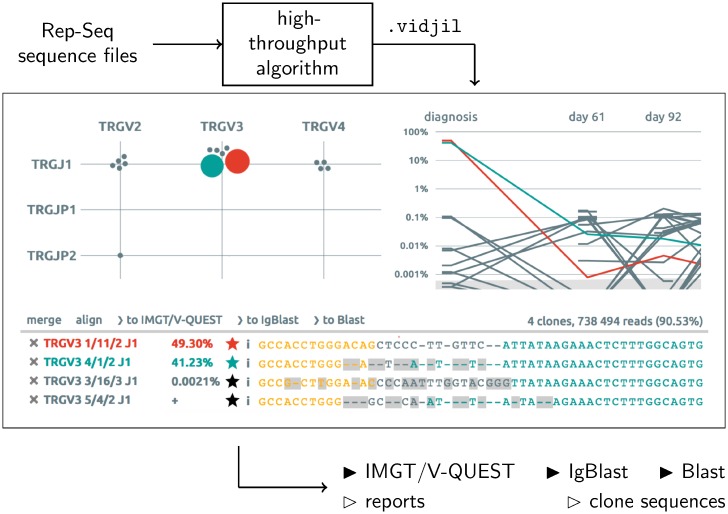
The Vidjil web application reads a .vidjil json file produced by a RepSeq algorithm gathering reads into clonotypes. The web application displays clonotypes on a grid (left), on a list with their representative sequences, possibly aligned (bottom), and on a time graph when there are several samples (right). Clonotypes can be annotated, edited or merged. Data can be exported or sent to other software. Thanks to the sample and patient/experiment database, the user directly uploads and processes her sequences from the web application and saves in the database her own edits and annotations.

The main components of the visualization are a list of clonotypes, a plot with either a grid view or a bar view, a window with the sequences and, when there are several samples, a graph (Fig 2). On the grid view, each clonotype is represented by a bubble. The axis of the grid can be changed (genes or alleles, N-diversity length, read length, CDR3 length, V distribution, similarity, GC content…). When the axis change, the bubbles move, using a collision detection implemented through a quad-tree [20].

**Fig 3:**
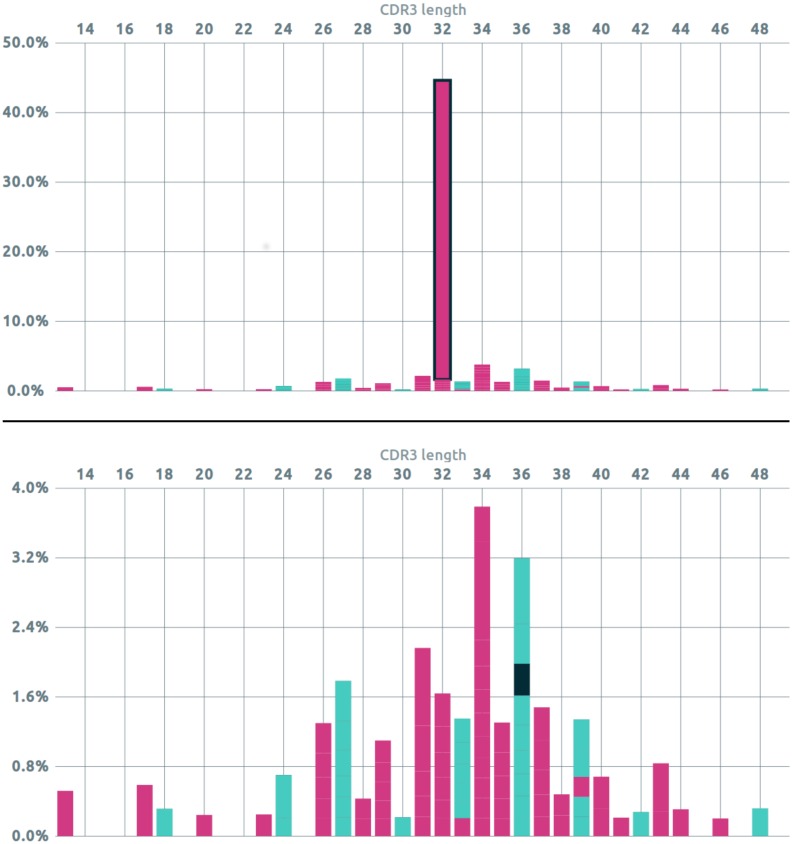
Interactive analysis of the CDR3 length distribution for the TR*γ* locus with the Vidjil web application. The publicly available L4 dataset is a diagnosis sample of an acute lymphoblastic leukemia patient (dataset and interactive visualization at vidjil.org/data). The color denotes productivity, magenta means the sequence is not productive, while light green is. Each portion of the bars represent clonotypes that can be interacted with. Top: There is a main peak at 32nt mainly constituted from the major (not productive) clonotype which prevents from correctly observing the remainder of the distribution. Bottom: After the main clonotype has been selected and hidden, the distribution can be more easily analyzed. The black portion of the bar at 36nt is hovered by the mouse. The related clonotype can be selected and further studied, *e.g.* by looking at its nucleotide sequence, sending to other analysis software, annotating it or filtering it.

Any click on a clonotype anywhere in the web application highlights the relevant elements in all the views, enabling to inspect clonotypes of interest and to further analyze or filter them. For instance one can plot the CDR3 distribution and remove the major clonotype to study in-depth the following ones (Fig 3).

The lower pane displays and aligns nucleic sequences, displaying additionnal data or features computed by the algorithm, such as V(D)J designation and CDR3 location. An user can modify the locus and V, D and J gene assignations made by the analysis software to correct any possible mis-assignation. The views will update accordingly.

Further manual or automatic clusterings can be explored. The user can merge similar clonotypes, that have differences coming from either biological (hypermutations) or technological (PCR, sequencing) reasons. To help the user making these merge decisions, the web application provides a multiple sequence alignment tool and a map based on the alignment distance between clonotypes using a tSNE algorithm [21]. The user can also decide to rely on the automatic merging tool, based on DBSCAN [22] which merges the clonotypes based on their sequence similarity and on their respective ratios: The smaller clonotypes can be merged into the bigger ones.

The user can further study or compare some clonotypes by automatically sending their sequences to IMGT/V-QUEST [3], IgBlast [6] or Blast [23] web forms. Some results of IMGT/V-QUEST (such as CDR3 sequence, productivity of the sequence, or boundaries of 5’V, D and 3’J) can be automatically retrieved with an asynchronous call to the IMGT API. The sequence features are then underlined in the interactive browser. Finally, a user can normalize the data relatively to the expected concentration of a clonotype that may be a spike of known concentration included in the sequenced samples. For instance, such normalizations may be used to quantify minimal residual disease.

### Server with Sample and Patient/Experiment Database

A sample database links the web application and the algorithmic part, allowing users to upload sequence files and manage their jobs directly from the web application. When uploading files, the user can choose some predefined preprocess to be launch on her data. At the moment we offer to merge paired-end reads using PEAR [24]. A server, implemented in Python with the web2py framework (web2py.com), queues the job requests, allowing many jobs to be scheduled without overloading the server.

Users may select the samples they want to display in the web application: either multiple samples from a unique patient made at different times (for example to study MRD or immune response), samples from different patients or experiments to compare their immune repertoires, or results made from the same sample with different biological or software pipelines.

The web application also generates printable reports that can be put in the patient’s medical record. These reports summarize the data either on a sample or for a collection of samples, giving information as the percentages of analyzed reads, the share of the different loci and the concentrations of the major clonotypes along the time.

The server has an authentication mechanism, ensuring data is only accessible to authorized users. By default, the files and the results are private, but they can also be shared to selected users or groups of users, or made public. Sensitive data such as personal information related to the patient can be kept private. Users in the same group may be granted different rights. Annotations and other edits made by authorized users can be saved. These annotations are added to the reports that the visualization tool can generate. The user can also export data to browse them offline, without any access to the server.

#### Server administration and maintenance

The Vidjil server is a full-stack environment, making use of many tools to ensure the system is healthy and suitable for use in a professional environment. Some of the notable features are: monitoring, regular backing-up of data, notifications to users for maintenances and upgrades. There are also many tools aimed at keeping a maintainable piece of software that provides satisfactory usage. The aforementioned tests are a part of these tools.

Installing the server in a hospital or a research lab does not require huge facilities (see usage or installation instructions in S1 File). In fact, disk storage to store input data (as for any high-throughput sequencing experiment) is the main constraint in our environment. Note that even when the input sequences are deleted, the server is still able to display the results of previous analyses. The computation requirements are very low: Our public test server (app.vidjil.org) runs with two Intel(R) Core(TM) i5-2400 CPU with 16GB RAM, characteristics that are now common even among laptops.

We provide Debian packages as well as Docker containers to ensure installing Vidjil is simple and fast. The goal is to make sure the overhead of setting up a Vidjil installation is as little as possible. With the material costs being so low, an automated packaging solution brings the time needed for installation down and therefore the cost.

## Results

### Clinical Data Analysis

We now illustrate what the Vidjil platform brought in RepSeq data analysis through two clinical usages of the application. Both the raw FASTQ files and the interactive visualization can be accessed at vidjil.org/data.

**Patient L3 (5 samples).** This dataset (patient 063 from [25]) contains one diagnosis sample and four follow-up samples of a patient with acute lymphoblastic leukemia (ALL). The patient received stem cell transplantation at day D183, and unfortunately relapsed at the end of the studied period. The libraries were prepared with PCR BIOMED-2 primers on the TR*γ* and IgH loci, and sequenced on an Ion Personal Genome Machine with an Ion 318 Chip Kit [25].

Results were analyzed on the web application using either Vidjil and MiXCR algorithm. Using the Vidjil web application with either algorithm enables to identify TR*γ* and IgH clonotypes and track them through the time, annotating specific clonotypes. In a routine practice, it allows to add further follow-up samples and continue the analysis.

Note that minor differences arise from Vidjil/MiXCR algorithms but the results are globally equivalent. Interestingly the relapse clonotype (day D308) on the TR*γ* locus, TRGV2*01 0/7/0 TRGJP1*01, was already observable at day D90 and above all at day D263 (second most abundant clonotype at this point, 1.3% of the reads). This clonotype was present at diagnosis on TR*γ*, but at a concentration two orders of magnitude below the main clonotype. However other clonotypes had a steady concentration all along the time while this clonotype had an increasing concentration. On the IgH locus, there is no similar clonotype: the main clonotypes at D308 were not detected in the diagnosis.

**Patient L4 (1 sample, with incomplete recombinations).** This dataset (patient 125 from [26]) contains one diagnosis sample coming from an ALL patient. The library was prepared with BIOMED-2 and custom TR*δ*, Ig*κ*, TR*γ* primers, and sequenced on an Ion Personal Genome Machine with an Ion 318 Chip Kit. This patient was included in a systematic comparison on 125 patients, some of which having incomplete D*δ*2-D*δ*3 and Intron-KDE recombinations [26].

This dataset shows both the ability of the algorithm to process these incomplete recombinations and the ability of the web application to display them. Moreover, the ability to process data with several analysis software inside the platform gives more confidence in the results. For example, IMGT/V-QUEST currently does not process Dd2-Dd3 and Intron-KDE recombinations, and thus do not process a large part of these clonotypes. However, running IgBlast or a plain Blast from the Vidjil web application on these clonotypes confirms the proposed recombinations.

### Usage of the Web Application

The public test server was opened in October 2014. 40 labs of 11 different countries submitted almost 14 billion sequences in 9,266 sequence files, with an average of 1.5M reads per file. Data mostly came from Illumina Mi-Seq and Ion Torrent sequencers. Paired-end data were entered either separately, or after processing by software such as PEAR [24] and pRESTO [27]. The Vidjil algorithm works on reads coming from either amplicon-based or capture-based deep sequencing strategy. While DNA-Seq sequencing with specific V(D)J primers usually lead to more than 95% analyzed sequences, capture with many probes or RNA-Seq strategies usually lead to datasets with less than 0.1% V(D)J recombinations.

Analysis times are compatible with daily research or clinical work. On a standard laptop, the version 2016.03 of the algorithm processes 1 Gbp in less than 5 minutes for a single locus. Multiple loci and incomplete recombinations require several iterations of the algorithm and may be up to 10× slower. On the server, 83% of the submitted jobs were processed in less than 10 minutes.

HTS offers the perspective of cheaper, quicker and more thorough analyses of patient lymphocytes. Vidjil has been designed with this purpose in mind: helping clinicians and researchers analyzing their data without further bioinformatics knowledge. Today, it is still the only available open-source RepSeq platform with a web application enabling this autonomous usage. Since the start of 2015, clinicians in the Lille hospital are routinely using HTS with Vidjil to study diagnosis of acute lymphoblastic leukemia (ALL) samples [26, 28] as well as chronic lymphocytic leukemia (CLL). Since June 2016, Paris Necker and Toulouse hospitals also routinely use the platform for ALL and CLL diagnosis. Several other hospitals also regularly use the platform (London, Brussels, Kiel, Bergamo, Montpellier to cite a few). Other Vidjil users have estimated the immunological repertoire in mice and rats [29, 30] and have evaluated the clonal diversity for the monitoring of minimal residual disease to better stratify ALL patients [31].

## Availability and Future Directions

### Availability

Vidjil is licensed under the GPLv3 open-source license. Source code, binaries and a public web server are available at vidjil.org and at bioinfo.lille.inria.fr/vidjil. Moreover, the sources are accessible within a software version control repository at git.vidjil.org alongside with the full code history. The algorithm can be used independently, like other RepSeq software, or through the web application, either on the public test server, or on a private server. Several hospitals are now installing in-house instances of the server.

Logging into the public server with the demo account give access to datasets showing features of the platform. The raw .fastq files of these datasets are also available on vidjil.org/data. Moreover, the curated sequences containing V(D)J recombinations can be obtained at vidjil.org/curated-vdj and could also be used for the calibration and the evaluation of other RepSeq software. Help is available from vidjil.org/doc and contains documentation on the algorithm, the web application, including a tutorial on the public datasets.

### Future Directions

The platform is constantly evolving. We regularly discuss with some of our users to see their needs and set future directions. A survey, conducted in September 2015, got answers from 17 labs around the world (results on vidjil.org/survey). A meeting in March 2016 for Vidjil users and developers gathered 35 people (vidjil.org/workshop-2016).

We continue to improve the algorithm, notably to process more incomplete or unusual recombinations. However, the design strategy of the whole platform is to give access to several algorithms and software. We already allow to process reads by either the Vidjil algorithm or MiXCR [10], that notably includes an error correcting method tailored to RepSeq data. We aim to package other open-source RepSeq algorithms through the platform. We also provide links to post-process selected clonotypes with IMGT/V-QUEST [3], IgBlast [6] or Blast [23]. Such post-processing may give further analysis, as with the IMGT/JunctionAnalysis detailed analysis of the CDR3/JUNCTION provided through IMGT/V-QUEST. Using IgBlast or Blast often helps to find low-identity similarities with some germlines genes or other regions, and can help to understand clonotype sequences with unusual recombinations, as explained above on the Patient L4.

The Vidjil platform is already scalable, as demonstrated by the more than 9,000 sequence files processed. Several hundreds of these samples were already processed in a routine hospital practice. In a production environment, special care should be taken on the disk space and on the backup methods. Installation of the server will be eased, both through improved packaging and more administration tools. To ease the operation in environments with hundreds of patients or experiments, the web application will soon be able to process files on remote storage servers through a mounted filesystem. Finally, as some labs or organizations are tuning Vidjil for their needs, we also enabled custom pre-processing in the platform through simple server configuration and we plan to do so also for post-processing.

New techniques in library preparation and in sequencing will require new analysis and visualization tools. Third-generation sequencing techniques and single cell sequencing may provide new insights on the immune repertoire. Different methodologies now allow to have access to informations on the receptor pairs [32–34]. Challenges for Vidjil, and, more generally, for other RepSeq software, include the handling of such pairs as well as new ways to algorithmically and interactively study whole immune repertoires.

## Supporting Information

S1 File Installation instructions and test data. This archive provides instructions for installing and using Vidjil. It also provides links to the test data as well as parameters used to launch the Vidjil algorithm.
(PDF)

S2 File Source code. The source code is also accessible on our source code repository (git.vidjil.org). Regular releases of the algorithm can be downloaded at vidjil.org/releases.
(TGZ)
