# Correction: Vidjil: A Web Platform for Analysis of High-Throughput Repertoire Sequencing

**DOI:** 10.1371/journal.pone.0172249

**Published:** 2017-02-09

**Authors:** Marc Duez, Mathieu Giraud, Ryan Herbert, Tatiana Rocher, Mikaël Salson, Florian Thonier

The affiliation for the sixth author is incorrect. The correct affiliation is: Inserm UMR1151, Institut Necker-Enfants Malades, Paris Descartes University and Necker–Enfants Malades Hospital–Paris, France.

There is also an error in the funding section. The current statement fails to mention that the author Florian Thonier (FT) is supported by Fondation EDF. The correct funding statement should read: This work was supported by SIRIC ONCOLille, Grant INCa-DGOS-Inserm 6041. FT is supported by Fondation EDF. The funders had no role in study design, data collection and analysis, decision to publish, or preparation of the manuscript.

## References

[1] Duez M, Giraud M, Herbert R, Rocher T, Salson M, Thonier F (2016) Vidjil: A Web Platform for Analysis of High-Throughput Repertoire Sequencing. PLoS ONE 11(11): e0166126 doi:10.1371/journal.pone.0166126 27835690 10.1371/journal.pone.0166126 PMC5106020

