# Supplementary material for: Vidjil: A Web Platform for Analysis of High-Throughput Repertoire Sequencing
Source: PLoS One. 2016 Nov 11;11(11):e0166126. doi: 10.1371/journal.pone.0166126 (PMC5106020; doi:10.1371/journal.pone.0166126)
Supplement: S1 File — This archive provides instructions for installing and using Vidjil. It also provides links to the test data as well as parameters used to launch the Vidjil algorithm. (PDF) [file pone.0166126.s001.pdf]

Vidjil: High-throughput analysis of immune repertoire
# Supplementary Material

## Installation and running instructions

**Web application usage, with server.** Two options are provided to run Vidjil.

- The test server `http://app.vidjil.org` is run by Université de Lille, SIRIC ONCOLille and University of Bristol as a service for the community. As disk space is shared by all the users, we set a limit of 50 GB per user. The detailed help of the web application, from the point of view of the user, is in the file `doc/browser.org`[1].
- The server can also be installed on a private network. Detailed instructions on how to install the server are provided in `doc/server.org`.

**Standalone usage, without server.** It is also possible to run locally the algorithm and the visualization, without connecting them to a server. The algorithm (C++) can be run from command line. Detailed compilation and run instructions are found in `doc/algo.org`. The algorithm outputs a `.vidjil` file that can directly be fed to the visualization (opened by loading the file `browser/index.html` in a web browser) in the `import/export` menu.

## Test data

The test data can be found at `http://vidjil.org/data`. Vidjil was launched on the dataset using the following parameters.

**Patient L3.** `-3 -z 100 -r 1 -g germline`

**Patient L4.** `-3 -z 100 -r 1 -g germline -i`
(note the additional `-i` for incomplete recombinations).

[1]These files are provided in the code archive sent with the submission, as well as on `http://www.vidjil.org`. Moreover, they are available online on the public git server.
